# Validity and reliability of the WHOQOL-BREF in a pregnant population

**DOI:** 10.1186/s12955-023-02166-2

**Published:** 2023-08-21

**Authors:** Elisabet Rondung, Sandra Oliveira, Francisco Esteves

**Affiliations:** 1 Department of Psychology and Social Work, Mid Sweden University, Östersund, 831 25 Sweden; 2 Faculdade de Ciências Humanas, Católica Research Centre for Psychological, Family, and Social Wellbeing, Universidade Católica Portuguesa, Lisbon, Portugal

**Keywords:** Quality of life, WHOQOL-BREF, Pregnancy, Women, Validity, Confirmatory factor analysis

## Abstract

**Background:**

Physical end emotional changes during pregnancy may not only affect pregnant womens’ quality of life, but also how instruments assessing quality of life perform in such populations. To date, there is insufficient evidence on psychometric properties for both generic and condition-specific instruments measuring quality of life during pregnancy. The aim of this study was thus to examine the structural validity, internal consistency, and construct validity of the WHOQOL-BREF in a sample of pregnant women.

**Methods:**

A convenience sample of 1015 pregnant women in Sweden completed the WHOQOL-BREF online. We examined the psychometric properties of the instrument using principal component analysis (PCA), confirmatory factor analysis (CFA), Cronbach’s alpha, item-domain correlations, correlations with overall QOL and general health, and multiple linear regression with items on overall QOL and general health as outcomes.

**Results:**

Principal Component Analysis in a random subsample (*n* = 502) supported a four-factor model, encompassing the domains physical, psychological, social and environmental quality of life, but with four of the items originally in the environmental domain relocated to the other domains. The proposed domain structure showed good fit in confirmatory factor analysis in the other random subsample (*n* = 513). The physical and psychological domains showed good internal consistency (Cronbach’s alpha = 0.885 and 0.826 respectively), while the social and environmental domains were weaker in this regard. All domains showed significant positive correlations with items on overall QOL and general health. The physical and psychological domains were the most evident predictors in the regression models.

**Conclusions:**

We find the Swedish version of the WHOQOL-BREF to have good psychometric properties to be used in samples of pregnant women, and propose an alternative domain structure that might be even more useful for assessing quality of life during pregnancy. The physical and psychological domains showed good internal consistency and construct validity.

Pregnancy is generally perceived as a period of transition, affecting women both physically and emotionally. Researchers have shown a dramatically increasing interest in women’s quality of life (QOL) during this period in life [1].

QOL has been conceptualized and defined in various ways. While health related QOL focuses on the individual’s perception of how their functioning and well-being is affected by their health status or a specific condition, generic QOL broadens the picture to encompass the individual’s satisfaction with life in general, not solely in relation to disease-related limitations on functioning [2]. The World Health Organization (WHO) defines QOL in this broad sense, as “individuals’ perception of their position in life in the context of the culture and value systems in which they live and in relation to their goals, expectations, standards and concerns” [3]. They highlight that QOL is a subjective experience of a multidimensional construct that pertains both positive and negative aspects of life. While there is no common understanding of the definite dimensions of QOL, most conceptualizations include physical, psychological and social dimensions [4].

The physical and emotional changes accompanying pregnancy may have an impact on womens’ QOL. Several studies have shown a generally lower level of QOL among pregnant women, when compared to non-pregnant women in the same age [5]. Above all, there seem to be an overall trend of decreasing physical QOL over the course of pregnancy, while psychological QOL often is shown to be stable or even increase [5]. Interestingly, Bai et al. [6] identified four different trajectories of QOL change throughout pregnancy: healthy (63%), consistently low (11%), small increase (13%) and large decrease (13%), suggesting individual variations from the group pattern.

With the different definitions of health related and generic QOL, instruments aimed to measure QOL also have their differences. Health related QOL can be measured either in a general way, such as in the SF-36 [7], but also using condition specific instruments. By including domains that are of central importance for a specific condition, these instruments are believed to be better equipped to capture small but important differences or changes in QOL in this particular group [8]. The downside of this specificity is that their use is limited to a particular patient group and to the individual’s perception of how they are affected by the specific health condition. In contrast to the disease-based paradigm, generic QOL instruments are designed to capture QOL in its broad sense, as an individual’s satisfaction with life regardless of potential health conditions [2]. These instruments comprise the individual’s subjective experience regarding multidimensional aspects of their life (e.g. self-esteem, body image, pain and discomfort, social relationships, and physical safety and security), in line with the pivotal definition of QOL by the WHO [3]. This personal view enables universal QOL comparisons between healthy and clinical groups, and repeated assessments of an individual’s QOL over different life stages. Hence, to compare QOL between pregnant and non-pregnant women, or study longitudinal changes in womens’ QOL over longer periods of time, we would need to use a generic measure of QOL. However, conditions associated with pregnancy might affect the validity of generic instruments during this specific period in life. Hence, generic instruments need to be validated in pregnant populations.

In a recent systematic scoping review, Brekke et al. [9] identified the use of twelve generic and seven specific QOL instruments in pregnant populations. One of the generic instruments that was most used was the World Health Organization’s Quality of Life-BREF (WHOQOL-BREF). It was developed by the WHOQOL Group as an abbreviated version of the WHOQOL-100 [10], reflecting physical, psychological, social, and environmental dimensions of QOL.

In their review, Brekke et al. [9] identified six studies that included some evaluation of the WHOQOL-BREF in a pregnant population [11–16]. Most simply reported Cronbach’s alpha of either the full scale (alpha = 0.84–0.92) [12–15] or specific domains (Physical: 0.64–0.86; Psychological: 0.58–0.78; Social: 0.44–0.77; and Environmental: 0.76–0.80) [11, 14–16]. The correlations between the physical, psychological and social domains were presented in a paper by Brandão et al. [11] (*r* = 0.461–0.527), while Vachkova et al. [16] and Mortazavi et al. [15] presented some basic descriptive statistics for the specific domains. Beyond that, psychometric evaluations of the WHOQOL-BREF were lacking. Similar or even sparser results were found for the other instruments identified. While these basic statistics might give a clue of scale reliability, proper psychometric evaluation requires a much more thorough approach [17]. As their overall conclusion, Brekke et al. [9] thus found the evidence on all psychometric properties insufficient, and strongly encouraged primary studies evaluating the psychometric properties for instruments that measure QOL during pregnancy.

In line with their suggestion, the aim of this study was to examine the psychometric properties of the WHOQOL-BREF, by evaluating its structural validity, internal consistency, and construct validity in a sample of pregnant women. If general measures are to be used to assess the QOL of pregnant women, it is imperative to understand how these instruments perform in such populations. Availability of a good, validated, generic QOL instrument would enable cross-sectional and longitudinal research, be useful as a validated patient-reported outcome measure in clinical trials, and facilitate clinical identification of women in need of support during pregnancy.

## Methods

This cross-sectional study was part of a project focusing on quality of life during pregnancy. Ethical approval was obtained from the regional ethical board in Umeå, Sweden (2018-451-31).

### Participants and procedure

Participants were self-recruited via an advertisement in social media (Facebook and Instagram). Interested individuals could follow a link to the online survey software Qualtrics, where the questionnaire was available from February 11 to March 11, 2019. Before starting the questionnaire, potential participants gave their digital informed consent to participate. The first items of the questionnaire confirmed that participants fulfilled the inclusion criteria, i.e. being at least 18 years of age and currently pregnant. No identifiable data was collected, and no exclusion criteria were adopted.

A total of 1016 women completed the questionnaire. One woman was excluded due to non-eligibility (low age), leaving a final sample of 1015 pregnant women.

The participating women had a mean age of 29.8 years (*SD* = 3.93). Most were in the second or third trimester of pregnancy. The vast majority were born in Sweden and lived together with a partner. Please see Table 1 for more participant details.

**Table 1:** Demographic and reproductive characteristics of the complete sample (n = 1015) and the two random subsamples

Characteristic	Complete sample (n = 1015)			PCA subsample (n = 502)			CFA subsample (n = 513)	
	*n*	%		*n*	%		*n*	%
**Parity**								
Have never been pregnant before	570	56.2		278	55.4		292	56.9
Have been pregnant, but not given birth	57	5.6		32	6.4		25	4.9
Have given birth before	388	38.2		192	38.2		196	38.2
1 previous birth (valid percent)	317	81.7		155	80.7		162	82.7
2 previous births (valid percent)	57	14.7		31	16.1		26	13.3
3 previous births (valid percent)	10	2.6		3	1.6		7	3.6
4 previous births (valid percent)	3	0.8		2	1.0		1	0.5
5 previous births (valid percent)	0	0.0		0	0.0		0	0.0
6 previous births (valid percent)	1	0.3		1	0.5		0	0.0
**Trimester**								
First trimester (gestational week 1–14)	101	10.0		49	9.8		52	10.1
Second trimester (gestational week 15–28)	345	24.0		170	33.9		175	34.1
Third trimester (gestational week 29 or more)	569	56.1		283	56.4		286	55.8
**Civil status**								
Married or cohabiting	980	96.6		481	95.8		499	97.3
Single or not living with partner	35	3.4		21	4.2		14	2.7
**Education**								
Primary school	18	1.8		7	1.4		11	2.1
High school	275	27.1		122	24.3		153	29.8
College or University (1–3 years)	296	29.2		143	28.5		153	29.8
College or University (more than 3 years)	426	42.0		230	45.8		196	38.2
**Occupational status** ^*^								
Employed	823	81.1		415	82.7		408	79.5
Self-employed	42	4.1		21	4.2		21	4.1
Student	93	9.2		39	7.8		54	10.5
On sick leave	125	12.3		56	11.2		69	13.5
On parental leave	67	6.6		38	7.6		29	5.7
On pregnancy leave	93	9.2		41	8.2		52	10.1
Unemployed	35	3.4		18	3.6		17	3.3
Other	7	0.7		6	1.2		1	0.2
**Country of birth**								
Sweden	955	94.1		469	93.4		486	94.7
Other	60	5.9		33	6.6		27	5.3
**Site of living**								
Large city	472	46.5		238	47.4		234	45.6
Medium sized town	272	26.8		127	25.3		145	28.3
Small town or rural area	271	26.7		137	27.3		134	26.1

Note. ^*^Several responses possible

### Measure

Participants responded to the Swedish version of the WHOQOL-BREF [10]. The instrument has 26 items where responses are given on a five-point Likert scale (range 1–5). Items 3–26 are used to derive four domain scores: physical health, psychological, social relationships, and environment [18]. Details on how the items are distributed between the domains can be found in Table 2. Items 1 and 2 are used as general measures of an individual’s perception of overall QOL (Q1) and general health (Q2). As suggested by Skevington et al. [19] the two general questions can also be added to a combined measure of overall quality of life and general health (Q1 + Q2). Although the WHOQOL-BREF has only been sparingly validated in pregnant populations, the instrument has been worldwide field-tested and its psychometric properties have demonstrated to be good to excellent, indicating it to be a valid instrument to be used across cultures and with a variety of population groups, in large epidemiological surveys, clinical settings and clinical trials [19].

**Table 2:** Structural validity: Factor loadings of the WHOQOL-BREF items in Principal Component Analysis with Varimax rotation

		Original domain	SS loading	Expl. variance	Communalities	Factor loadings			
						Phy	Psy	Soc	Env
**Physical QOL**			4.73	19.69%					
3	Constrains by physical pain	Phy			0.684	**0.816**	0.002	0.073	0.113
4	Dependency on medical treatment	Phy			0.382	**0.582**	0.207	0.001	0.024
10	Energy	Phy			0.630	**0.701**	0.356	0.102	0.031
14	Opportunity for leisure activities	Env			0.562	**0.648**	0.205	0.283	0.139
15	Mobility	Phy			0.655	**0.786**	− 0.044	0.173	0.075
16	Satisfaction with sleep	Phy			0.459	**0.599**	0.256	− 0.038	0.184
17	Activities of daily living	Phy			0.738	**0.809**	0.255	0.100	0.091
18	Work capacity	Phy			0.643	**0.741**	0.286	0.088	0.068
**Psychological QOL**			4.17	17.38%					
5	Enjoy life	Psy			0.693	0.201	**0.757**	0.280	− 0.011
6	Meaningful life	Psy			0.687	0.120	**0.765**	0.296	− 0.007
7	Concentration	Psy			0.547	0.396	**0.590**	− 0.088	0.185
8	Safety in daily life	Env			0.548	0.192	**0.616**	0.029	0.361
9	Healthy physical environment	Env			0.237	0.082	**0.418**	0.171	0.162
11	Acceptance of bodily appearance	Psy			0.267	0.087	**0.403**	0.101	0.294
19	Satisfaction with oneself	Psy			0.619	0.335	**0.643**	0.243	0.184
26	Absence of negative feelings	Psy			0.586	0.265	**0.690**	0.092	0.175
**Social QOL**			2.03	8.47%					
20	Personal relationships	Soc			0.606	0.201	0.534	**0.524**	0.078
21	Satisfaction with sex life	Soc			0.466	0.272	0.240	**0.553**	− 0.171
22	Satisfaction with support from friends	Soc			0.443	0.070	0.277	**0.572**	0.183
23	Satisfaction with home environment	Env			0.435	− 0.011	0.149	**0.577**	0.282
**Environmental QOL**			2.20	9.16%					
12	Financial resources	Env			0.608	0.067	0.357	− 0.062	**0.687**
13	Availability of information	Env			0.486	0.137	0.216	0.051	**0.646**
24	Access to health services	Env			0.594	0.135	0.103	0.380	**0.649**
25	Satisfaction with access to transport	Env			0.556	0.151	− 0.067	0.465	**0.559**

*Note*. Phy = Physical domain; Psy = Psychological domain; Soc = Social domain; Env = Environmental domain.

### Statistical analyses

Statistical analyses were guided by the COSMIN design and reporting standards [20, 21], the WHOLQOL-BREF manual [18] and the original WHOQOL-BREF validation [19].

All cases had complete data. The dataset was split in two random samples (*n* = 502 and *n* = 513), using the function “random sample of cases” in SPSS. Participant characteristics were computed for the complete sample as well as for the two subsamples, and the samples were compared using a one-way ANOVA (regarding the four original domains of the WHOQOL-BREF, items 1 and 2 separately, age, and gestational week) and Pearson’s Chi-Square tests (regarding parity, trimester, civil status, education, occupational status, country of birth, and site of living). No significant differences were identified between the two subsamples.

To assess the structural validity, we first explored the factor structure of items 3–26 using Principal Component Analysis (PCA) with Varimax rotation in one of the random subsamples (the PCA-sample, *n* = 502). The Kaiser-Meier Olkin (KMO) measure and Bartlett’s test of sphericity were inspected to verify the adequacy of the sampling and the correlation structure. The resulting model was examined with regard to its initial eigenvalues, the sum of squared loadings and variance explained by each factor after rotation, and the communalities and cross loadings of each item. As suggested by Costello and Osborne [22], communalities lower than 0.40, loadings lower than 0.32, and cross-loadings of 0.32 or higher were considered as problematic.

The PCA was followed by a confirmatory factor analysis (CFA) of the identified factor structure in the other random sample (CFA sample, *n* = 513), using diagonal weighted least squares (DWLS) as estimator [23]. The goodness of fit was evaluated using a combination of methods; the normed Chi Square (*χ*^*2*^*/df*) where values lower than 2, or in more generous recommendations lower than 5, have been suggested acceptable [24], the Comparative Fit Index (CFI) where values ≥ 0.95 suggest good fit, the Root Mean Square Error of Approximation (RMSEA) where values ≤ 0.06 indicate a good fit and vales ≤ 0.08 could suggest an acceptable fit especially if the upper limit of the 90% confidence interval falls below this threshold, and finally the Standardized Root Mean Square Residual (SRMR) where 0.08 indicates a good fit [24–26]. We then inspected the modification indices to explore if the model fit could be improved by allowing item residuals with high covariance to correlate. Pairs of correlating residuals were added to the model one by one, beginning with the ones with highest covariance, until new additions only made minor changes in the fit indices. Residuals were only allowed to correlate if the items belonged to the same domain and correlations appeared theoretically meaningful. For comparative purposes, we also tested the fit of the original four domains, both in a basic model and in a model with correlated residuals, applying the same procedure as described above.

Internal consistency of both the original and proposed domains was assessed using Cronbach’s alpha, alpha if item was deleted, and item-domain correlations. A Cronbach’s alpha of 0.70 or higher and item-domain correlations of 0.30 or higher were considered adequate [27].

As a preliminary test of construct validity, we calculated Person’s correlations (one-tailed) between both original and proposed domains and overall QOL and general health (Q1, Q2 and Q1 + Q2). We also conducted multiple linear regression analyses (enter method) with overall QOL and general health as outcomes, using the complete sample (*n* = 1015). Separate analyses were conducted using the original and proposed domains as predictors. Domain scores were calculated by the mean of the included items multiplied by 4 [18]. All domains were expected to show a significant positive correlation with the general items (Q1, Q2, Q1 + 2), and especially strong correlations were expected between the physical domain and the general health item (Q2), and between the psychological domain and overall QOL item (Q1). We also expected to see a unique and positive contribution of each domain in the multiple regression models. Correlations were interpreted as suggested by Dancey and Reidy: *r* = 0.10–0.39 indicating a weak correlation, *r* = 0.40–0.69 indicating a moderate correlation, and *r* ≥ 0.70 indicating a strong correlation [28].

All statistical analyses, except the CFA, were conducted in SPSS, version 27. The CFA was conducted using JASP, version 0.16.1.

## Results

### Structural validity

In the PCA subsample, The KMO measure verified the sampling adequacy (KMO = 0.917) and a significant result of the Bartlett’s test of sphericity (p < .001) indicated adequacy of the correlation structure. The ratio of participants per item was 1:21, which is in line with the thresholds of sample size recommendations [22]. Inspection of the eigenvalues indicated a possible fifth factor with an initial eigenvalue just above one (1.040). However, we found this factor neither theoretically nor statistically convincing, as one of its two items cross-loaded with the psychological domain and both items saturated with their original domain (psychological QOL) in a four-factor solution.

After rotation, the four-factor solution explained 54.71% of the total variance. The factor structure, including the sum of squared loadings and variance explained by each factor, is shown in Table 2. The identified factors showed an overall resemblance with the original domains of the WHOQOL-BREF [19], leading us to use the same domain names, that is, physical, psychological, social, and environmental QOL. However, factor loadings suggested that four of the items that originally were part of the environmental domain related more strongly to other factors in this sample (item 14 with the physical domain, items 8 and 9 with the psychological domain, and item 23 with the social domain). All items had a factor loading > 0.32 in their respective new domain. Three items (items 4, 9 and 11) showed low communalities (< 0.40) and eight items (items 7, 8, 10, 12, 19, 20, 24, and 25) had cross loadings larger than 0.32. With the intention to validate the original scale, we decided to retain all items, and rather identify and discuss their shortcomings in the current sample.

In the confirmatory factor analysis, we tested the factor structure proposed by the PCA in another sample (*n* = 513). Factor loadings are displayed in Table 3 and fit indices in Table 4. Fit indices of the proposed domain model indicated a good model fit, with CFI > 0.95 and SRMR < 0.08. Although the RMSEA did not reach below 0.06, the upper limit of the 90% CI was below 0.08 indicating acceptable fit. Fit indices were further improved after consulting the modification indices, and one by one allowing residual covariances to correlate. With four correlating pairs of residuals, RMSEA was below the 0.06 threshold. All factor loadings were acceptable (> 0.32) both in the basic model and in the model with correlated residuals (see Table 3).

**Table 3:** Structural validity: Factor loadings of the WHOQOL-BREF items in Confirmatory factor analyses (DWLS)

	Original domains Basic model					Original domains Correlated residuals^a^					Proposed domains Basic model					Proposed domains Correlated residuals^b^			
Item	Phy	Psy	Soc	Env		Phy	Psy	Soc	Env		Phy	Psy	Soc	Env		Phy	Psy	Soc	Env
3	0.764					0.700					0.764					0.681			
4	0.607					0.613					0.608					0.581			
10	0.774					0.778					0.771					0.777			
14				0.753					0.747		0.706					0.714			
15	0.771					0.703					0.770					0.703			
16	0.623					0.628					0.623					0.630			
17	0.909					0.920					0.900					0.913			
18	0.842					0.846					0.841					0.846			
5		0.910					0.806					0.902					0.798		
6		0.801					0.675					0.792					0.667		
7		0.651					0.653					0.640					0.647		
8				0.780					0.778			0.730					0.743		
9				0.501					0.498			0.470					0.475		
11		0.490					0.490					0.483					0.487		
19		0.826					0.827					0.815					0.823		
26		0.711					0.711					0.702					0.706		
20			0.860					0.860					0.853					0.854	
21			0.478					0.478					0.476					0.475	
22			0.649					0.649					0.648					0.647	
23				0.491					0.487				0.573					0.573	
12				0.486					0.483					0.590					0.581
13				0.620					0.617					0.760					0.750
24				0.523					0.466					0.648					0.575
25				0.536					0.483					0.672					0.601

*Note*. ^a^The residuals of items 5–6, 24–25, and 3–15 were allowed to correlate; ^b^The residuals of items 5–6, 3–15, 24–25, and 3–4 were allowed to correlate; Phy = Physical domain; Psy = Psychological domain; Soc = Social domain; Env = Environmental domain.

**Table 4:** Structural validity: Fit indices of models tested in Confirmatory factor analysis (DWLS)

Fit indices	Original domains Basic model	Original domains Correlated residuals^a^	Proposed domains Basic model	Proposed domains Correlated residuals^a^
Chi Square (χ^2^)	1319.464^***^	1028.192^***^	942.682^***^	667.919^***^
*df*	246	243	246	242
Normed Chi Square (χ^2^/*df*)	5.364	4.231	3.832	2.760
CFI	0.964	0.973	0.976	0.986
RMSEA (90% CI)	0.092 (0.087–0.097)	0.079 (0.074–0.084)	0.074 (0.069–0.079)	0.059 (0.053–0.064)
SRMR	0.085	0.078	0.073	0.064

*Note*. *df* = degrees of freedom; CFI = Comparative Fit Index; RMSEA = Root Mean Square Error of Approximation; CI = Confidence Interval; SRMR = Standardized Root Mean Square Residual; ^a^The residuals of items 5–6, 24–25, and 3–15 were allowed to correlate; ^b^The residuals of items 5–6, 3–15, 24–25, and 3–4 were allowed to correlate; ^***^ p < .001.

For comparative purposes, we also tested the fit of the original domain structure. Although showing relatively good fit indices too, the original model had a poorer fit with our data than the proposed model had (see Table 4).

### Internal consistency

Cronbach’s alpha for the complete scale was 0.90 (0.91 in the PCA sample and 0.90 in the CFA sample respectively). The alpha values of the original and the proposed four domains are presented in Table 5, alongside alpha values if items were deleted and the corrected item-total correlations. The physical and psychological domains showed high internal consistency in both solutions, the social domain was below 0.70 in both, and the environmental domain reached above 0.70 in the original whilst not in the proposed domain structure. Cronbach’s alpha if items were deleted indicated some issues with items 11 and 21 in both domain structures, and also item 23 in the proposed structure. Item-domain correlations supported a general pattern of higher internal consistency in the physical and psychological domains and lower correlations in the social and environmental domains. All item-domain correlations were above 0.30. Five correlations in the original domains solution and four in the proposed domain solution were weak (< 0.40).

**Table 5:** Internal consistency: Cronbach’s alpha and item-domain correlations for the original and proposed four-factor domains (n = 1015)

	Original 4-domain structure			Proposed 4-domain structure	
	Cronbach’s alpha (alpha if item deleted)	Corrected item-domain correlations		Cronbach’s alpha (alpha if item deleted)	Corrected item-domain correlations
**Physical domain**	**0.874**			**0.885**	
Item 3	(0.848)	0.711		(0.865)	0.709
Item 4	(0.873)	0.516		(0.885)	0.509
Item 10	(0.857)	0.647		(0.870)	0.670
Item 14^a^	-	-		(0.874)	0.628
Item 15	(0.855)	0.666		(0.869)	0.679
Item 16	(0.871)	0.532		(0.882)	0.542
Item 17	(0.837)	0.796		(0.856)	0.803
Item 18	(0.846)	0.720		(0.864)	0.718
**Psychological domain**	**0.803**			**0.826**	
Item 5	(0.749)	0.686		(0.789)	0.687
Item 6	(0.761)	0.628		(0.795)	0.644
Item 7	(0.790)	0.477		(0.811)	0.510
Item 8^a^	-	-		(0.798)	0.609
Item 9^a^	-	-		(0.827)	0.361
Item 11	(0.831)	0.380		(0.840)	0.382
Item 19	(0.737)	0.705		(0.785)	0.686
Item 26	(0.763)	0.602		(0.796)	0.619
**Social domain**	**0.612**			**0.646**	
Item 20	(0.376)	0.530		(0.499)	0.544
Item 21	(0.658)	0.342		(0.651)	0.349
Item 22	(0.515)	0.420		(0.545)	0.472
Item 23^a^	-	-		(0.612)	0.373
**Environmental domain**	**0.747**			**0.663**	
Item 8^a^	(0.707)	0.523		-	-
Item 9^a^	(0.730)	0.393		-	-
Item 12	(0.714)	0.485		(0.644)	0.403
Item 13	(0.714)	0.507		(0.596)	0.474
Item 14^a^	(0.737)	0.381		-	-
Item 23^a^	(0.731)	0.384		-	-
Item 24	(0.712)	0.488		(0.560)	0.501
Item 25	(0.719)	0.449		(0.589)	0.454

*Note.*^a^ = Originally in the Environmental domain

### Construct validity

Pearson’s correlations, displayed in Table 6, showed that all original and proposed domains correlated positively with each other and with the items on overall QOL and general health (*p* < .001). The physical and psychological domains showed moderate correlations with the overall items (*r* = 0.55–0.68), the social domain displayed weak to moderate correlations (*r* = 0.38–0.47), and the environmental domain weak correlations in the proposed structure (*r* = 0.29–0.33) whilst moderate when using the original domain structure (*r* = 0.43–0.49).

**Table 6:** Construct validity: Pearson’s r correlations between domains and overall QOL and general health (n = 1015)

	Q1	Q2	Q1 + Q2	Proposed Phy	Proposed Psy	Proposed Soc	Proposed Env
Overall QOL (Q1)	-	0.667^***^	0.906^***^	0.555^***^	0.636^***^	0.465^***^	0.314^***^
Overall health (Q2)	0.667^***^	-	0.920^***^	0.586^***^	0.567^***^	0.384^***^	0.289^***^
Overall QOL and health (Q1 + Q2)	0.906^***^	0.920^***^	-	0.625^***^	0.657^***^	0.463^***^	0.330^***^
Physical domain (Original)	0.545^***^	0.579^***^	0.616^***^	-	0.552^***^	0.372^***^	0.365^***^
Psychological domain (Original)	0.627^***^	0.574^***^	0.656^***^	0.544^***^	-	0.586^***^	0.490^***^
Social domain (Original)	0.457^***^	0.382^***^	0.458^***^	0.370^***^	0.565^***^	-	0.403^***^
Environmental domain (Original)	0.477^***^	0.425^***^	0.493^***^	0.496^***^	0.593^***^	0.493^***^	-

*Note.* Correlations with Original domains are shown under the diagonal, and correlations with the proposed domains above the diagonal; ^***^ p < .001.

When entering the domains simultaneously into multiple linear regression models (see Table 7), the physical and above all the psychological domains were strong independent predictors of overall QOL and general health. The social domain contributed significantly and positively to overall QOL (Q1) and the composite outcome (Q1 + Q2), whilst not to general health (Q2). The environmental domain showed different patterns when using the original and proposed items; in the first case showing small and only occasionally significant positive associations with all three outcomes, in the second case again showing small and marginally significant, but negative associations with all three outcomes.

**Table 7:** Construct validity: Standardized Beta coefficients (β) and Adjusted R^*2*^ *from multiple linear regression analyses, (n = 1015)*

	Outcomes		
	Overall QOL (Q1)	Overall health (Q2)	Overall QOL and health (Q1 + Q2)
**Original domains**			
Adj. R^2^	0.46	0.43	0.53
Predictors			
Original Phy	0.264^***^	0.371^***^	0.350^***^
Original Psy	0.382^***^	0.334^***^	0.391^***^
Original Soc	0.111^***^	0.047 ^ns^	0.085^**^
Original Env	0.065^*^	0.020 ^ns^	0.045 ^ns^
**Proposed domains**			
Adj. R^2^	0.47	0.43	0.54
Predictors			
Proposed Phy	0.291^***^	0.394^***^	0.377^***^
Proposed Psy	0.426^***^	0.337^***^	0.416^***^
Proposed Soc	0.128^***^	.057^ns^	0.100^***^
Proposed Env	− 0.053^*^	− .043^ns^	− 0.052^*^

Note. ^***^ p < .001; ** p < .01; ^*^ p < .05; ^ns^ non-significant

## Discussion

In this study, we sought to evaluate the structural validity, internal consistency, and construct validity of the WHOQOL-BREF in a sample of pregnant women. At a general level, all analyses supported a four-factor model, encompassing the domains physical, psychological, social, and environmental QOL. However, the PCA suggested an alternative distribution of items between these domains, with four of the items originating in the environmental domain [19] relocated to the other domains. Although the CFA revealed a relatively good fit of the original domain structure, the proposed model structure was superior in all fit indices, indicating the proposed distribution of items to be more adequate in this sample of pregnant women.

In further comparisons between the original and proposed domain structures, the general pattern of findings suggested that the physical, psychological and social domains worked slightly better in the proposed structure, whilst the environmental domain performed better in its original eight-item version. The physical and psychological domains showed good internal consistency in both models, with a Cronbach’s alpha that was higher than in other studies of pregnant populations [11, 14–16]. However, just like the majority of centers in the original validation by Skevington et al. [19], the alpha values of domains with fewer items were lower. In our case, the social and the proposed four-item environmental domains only reached marginal alpha values. The social domain has systematically presented marginal levels of internal consistency [19, 29–31], also in pregnant samples [11, 14, 16]. Despite the low alpha values, each item had strong factor loadings and contributed to the reliability (as inspected by alpha if item deleted). However, the alpha statistics of the proposed four-item environmental domain was lower than typically shown in pregnant samples [11, 14–16]. Although the lower internal consistency of these domains might be partly explained by the low number of items in these domains [32], the relatively low item-domain correlations further questions the consistency of these domains.

The relocation of item 14 was found both relevant and statistically successful. Physical limitations during pregnancy might well restrict the individual’s opportunities to take part in leisure activities. Interestingly, Taylor et al. [33] also found item 14 to load on the physical domain when validating the WHOQOL-BREF in people with rheumatoid arthritis, which further supports this hypothesis. In our study, the item performed well in its new domain, with high loadings in the CFA and higher item-domain correlations when compared to its original placing in the environmental domain.

Item 8, asking about safety in daily life, was found to be strongly associated to both the environmental and psychological domains. Although cross-loading on the environmental domain, the PCA showed a clear belonging in the psychological domain which was supported by higher item-domain correlations in the proposed domain structure. As the Swedish word for “safe” used in the translation have strong connotations of psychological safety, this double loading seem reasonable. Neither is it unique to this study. In the original cross-cultural validation of the scale, this item correlated more strongly with the psychological domain in seven of the 24 countries represented [19], and high loadings on this domain have been found in other validation studies [29, 34].

Items 9 (physical environment) and 23 (conditions of living place) could best be described as generally problematic in this sample, with low communalities and factor loadings regardless of domain. Item 9 has no apparent connection to psychological QOL, and the loading on that domain was surprising to us. One hypothesis might be that its place in the questionnaire, following three psychological questions and item 8 on safety in daily life, might have primed the participants to follow a similar response pattern. Another could be that an unhealthy environment leads to worry, associating the item with other issues of a psychological nature. With regard to item 23, our guess would be that it its focus on the domestic parts of life might explain its association to the social domain. With indications that Cronbach’s alpha would decrease rather than increase if deleted, the item appeared to make a significant contribution to its new domain.

As hypothesized, all domains showed significant positive correlations with the items on overall QOL and general health. As expected and in line with previous research [19, 30], the physical domain was the strongest predictor of general health (Q2) and the psychological domain was the strongest predictor or overall QOL (Q1) and also the compound variable of Q1 + Q2. While the contribution of the social domain was smaller, this domain made a positive and significant prediction of the outcomes including overall QOL (Q1 and Q1 + Q2), but could not predict general health (Q2). The environmental domain was the weakest predictor of all outcomes, regardless of model. Its non-significant or week associations with the general items suggest that specific environmental issues (money, access to information, health services, and transports) play a minor role in determining the overall QOL and general health of pregnant women in a high-income context such as the Swedish, while other environmental factors may assume a psychological and social dimension in pregnant women. As suggested by Xia et al. [31], the environmental domain might be context rather than health related.

### Limitations

This study was conducted in a convenience sample of self-recruited pregnant women, which might pose a risk to the representativeness of the sample and the generalizability of results. With the sample being rather large, and representing women of different parities, trimesters, educational levels, countries of birth, and sites of living, we believe that the findings still might be generalizable to the general population of pregnant women living in Sweden. It is also worth mentioning that we collected our data before the outbreak of the COVID-19-pandemic and the additional challenges faced by pregnant women under these difficult circumstances.

The study might have benefited from having a comparative sample of non-pregnant women of the same age. Unfortunately, no such data was available to us.

## Conclusion

In this study, the Swedish version of the WHOQOL-BREF was found to have good psychometric properties to be used in samples of pregnant women. While the original domain structure showed acceptable fit, we propose an alternative domain structure with even better fit that might be more useful for assessing QOL in pregnant samples. The physical and psychological domains showed good internal consistency and construct validity, while some uncertainty remains regarding the social and environmental domains. Future studies of QOL in pregnant populations are needed to evaluate the usefulness of this or other alternative domain structures.

## Data Availability

The datasets used and/or analysed during the current study are available from the corresponding author on reasonable request.
